# Combined Omics Reveals That Disruption of the Selenocysteine Lyase Gene Affects Amino Acid Pathways in Mice

**DOI:** 10.3390/nu11112584

**Published:** 2019-10-26

**Authors:** Lucia A. Seale, Vedbar S. Khadka, Mark Menor, Guoxiang Xie, Ligia M. Watanabe, Alexandru Sasuclark, Kyrillos Guirguis, Herena Y. Ha, Ann C. Hashimoto, Karolina Peplowska, Maarit Tiirikainen, Wei Jia, Marla J. Berry, Youping Deng

**Affiliations:** 1 Department of Cell and Molecular Biology, John A. Burns School of Medicine, University of Hawaii, Honolulu, HI 93813, USA; ligiamw@hawaii.edu (L.M.W.); asasu@hawaii.edu (A.S.); kyrillos@hawaii.edu (K.G.); hyh231@hawaii.edu (H.Y.H.); ahashimo@hawaii.edu (A.C.H.); mberry@hawaii.edu (M.J.B.); 2 Department of Quantitative Health Sciences, Bioinformatics Core Facility, John A. Burns School of Medicine, University of Hawaii, Honolulu, HI 96813, USA; vedbar@hawaii.edu (V.S.K.); mmenor@hawaii.edu (M.M.); dengy@hawaii.edu (Y.D.); 3 Cancer Biology Program and Metabolomics Shared Resource, University of Hawaii Cancer Center, University of Hawaii, Honolulu, HI 96813, USA; gxie@cc.hawaii.edu (G.X.); wjia@cc.hawaii.edu (W.J.); 4 Population Sciences in the Pacific Program and Genomics and Bioinformatics Shared Resource, University of Hawaii Cancer Center, University of Hawaii, Honolulu, HI 96813, USA

**Keywords:** metabolomics, selenium, transcriptomics, liver, selenocysteine, lyases

## Abstract

Selenium is a nonmetal trace element that is critical for several redox reactions and utilized to produce the amino acid selenocysteine (Sec), which can be incorporated into selenoproteins. Selenocysteine lyase (SCL) is an enzyme which decomposes Sec into selenide and alanine, releasing the selenide to be further utilized to synthesize new selenoproteins. Disruption of the selenocysteine lyase gene (*Scly*) in mice (*Scly^−/−^* or Scly KO) led to obesity with dyslipidemia, hyperinsulinemia, glucose intolerance and lipid accumulation in the hepatocytes. As the liver is a central regulator of glucose and lipid homeostasis, as well as selenium metabolism, we aimed to pinpoint hepatic molecular pathways affected by the *Scly* gene disruption. Using RNA sequencing and metabolomics, we identified differentially expressed genes and metabolites in the livers of Scly KO mice. Integrated omics revealed that biological pathways related to amino acid metabolism, particularly alanine and glycine metabolism, were affected in the liver by disruption of Scly in mice with selenium adequacy. We further confirmed that hepatic glycine levels are elevated in male, but not in female, Scly KO mice. In conclusion, our results reveal that Scly participates in the modulation of hepatic amino acid metabolic pathways.

## 1. Introduction

The trace element selenium is classically known for being critical to enhancing the efficiency of several redox reactions, attributed to the presence of the selenium-containing amino acid selenocysteine (Sec), an integral part of a group of proteins called selenoproteins [1]. Sec can be acquired through the diet or as a result of selenoprotein degradation, when Sec is decomposed through selenium recycling systems and resynthesized for insertion in the primary structure of newly formed selenoproteins [2].

Sec decomposition is catalyzed by the enzyme, selenocysteine lyase (SCL according to Protein Data Bank; EC:4.4.1.16; mSCL for the murine SCL), resulting in the release of selenide and alanine [3]. Selenide is postulated to be delivered back to selenoprotein synthesis [4]. *Scly* gene expression and mSCL expression and activity are highest in the kidneys and liver. The liver, in particular, is a major storage site for selenium and the site where selenium metabolism is coordinated in vertebrates. When dietary selenium is limiting, hepatic Scly is upregulated in mice [5], possibly contributing to maintaining selenoprotein synthesis. The liver also integrates central pathways in energy homeostasis, coordinating carbohydrate, lipid, and amino acid metabolism.

Interestingly, we have found that disruption of the *Scly* gene in mice led to obesity with dyslipidemia, hyperinsulinemia, glucose intolerance, and lipid accumulation in hepatocytes, characteristics of a metabolic syndrome-like phenotype. Scly KO mice also presented a localized selenium deficiency in the liver, even when fed a selenium-adequate diet [6]. Because of the effects of *Scly* disruption on energy metabolism, particularly insulin signaling [6,7], we sought to determine the pathways most affected and possibly responsible for the phenotype displayed by this mouse model.

In this study, we performed RNA Sequencing (RNA-Seq) analysis to determine differentially expressed genes in the livers of male Scly KO mice fed selenium adequate or selenium-deficient diets. Given the previously observed lipid deposition in the livers of Scly KO mice under low dietary selenium conditions, we expected differences between Scly KO and WT mice to be enhanced by low dietary selenium. Furthermore, we used a combined transcriptomics and metabolomics approach to identify hepatic molecular pathways influenced by the disruption of *Scly*-dependent selenium recycling in mice. Using this approach, we identified alterations in amino acid metabolism after the *Scly* gene disruption. Additionally, we pinpointed glycine as a differential amino acid elevated in the livers of Scly KO mice in a sex-dependent manner.

## 2. Materials and Methods

### 2.1. Animals and Diets

Mice lacking the *Scly* gene (Scly KO) were housed as a homozygous colony in our Animal Vivarium with a 12 h of light/dark cycle at 23 °C. C57BL/6N wild-type (WT) mice were obtained from The Jackson Laboratories and bred in-house for at least 20 generations. Weanlings were started on customized diets containing either 0.08 μg/g (mildly low) or 0.25 μg/g (adequate) of selenium as sodium selenite. Mice remained on these defined selenium diets *ad libitum* for at least eight weeks prior to euthanasia by CO_2_ asphyxiation. Livers and serum were removed after euthanasia and either snap-frozen or placed in RNAlater (Thermo Fisher Scientific, Waltham, MA, USA). Animal procedures were performed according to the National Institutes of Health Guide for the Care and Use of Laboratory Animals and approved by the Institutional Animal Care and Use Committee of the University of Hawaii, protocol n. 17-2616.

### 2.2. Reagents and Antibodies

Unless otherwise stated, all reagents were purchased from Sigma-Aldrich/MilliporeSigma (Burlington, MA, USA). Antibodies used were mouse monoclonal anti-DMGDH (Santa Cruz Biotechnology, Dallas, TX, USA, catalog n. sc-393178) and anti-alpha-tubulin (Novus Biologicals, Centennial, CO, USA, catalog n. NB100-690).

### 2.3. RNA Extraction and Sequencing

Liver tissues were disrupted with disposable probes using the Qiagen TissueRuptor, and total RNA was extracted using Qiagen AllPrep DNA/RNA Kit (Qiagen, Germantown, MD, USA). The quality of RNA samples was evaluated with Agilent BioAnalyzer (Agilent, Santa Clara, CA, USA) using the Nano RNA Kit. Five hundred ng of total RNA was used for mRNA isolation with the NEBNext^®^ Poly(A) mRNA Magnetic Isolation Module (New England Biolabs, Ipswich, MA, USA). Libraries for Next Generation Sequencing were prepared using NEBNext Ultra II Directional RNA Library Prep Kit for Illumina (New England Biolabs) following the manufacturer instructions. The quality of the libraries was evaluated with the Agilent BioAnalyzer using High Sensitivity DNA chips. To ensure optimal cluster density, the libraries were quantified with qPCR using KAPA/Roche Library Quantification Kit. Libraries were normalized, pooled, denatured and loaded on the flow cell at the 1.8 pM concentration. Sequencing was conducted with Illumina NextSeq 500 platform High Output/300 cycle kits (Illumina, San Diego, CA, USA).

### 2.4. Quantitative PCR (qPCR)

One microgram of total RNA was reverse-transcribed using High Capacity cDNA Reverse Transcription Kits (Applied Bioscience/ Thermo Fisher Scientific, Waltham, MA, USA), with 10 ng of resulting cDNA used for qPCR with PerfeCTa SYBR Green FastMix (Quantabio, Beverly, MA, USA) and 45 amplification cycles, in a 384-well plate platform of a LightCycler 480 II (Roche, Basel, Switzerland). Relative quantification used the ∆^−CT^ method, normalized to either hypoxanthine-guanine phosphoribosyltransferase (*Hprt1*), beta-actin (*Actb*) mRNA or 18S rRNA levels. Reported results used *Actb* expression in females and *Hprt1* in males as housekeeping genes for relative quantification. Table S1 provides primer sequences used in this study. All primers were evaluated for their efficiency prior to use in experiments

### 2.5. Metabolomics Analysis

Standards (approximately 1200 mammalian metabolites) were acquired from Sigma-Aldrich/MilliporeSigma, Santa Cruz Biotechnology, or Avanti Polar Lipids (Alabaster, AL, USA). The standards were prepared in appropriate solutions and analyzed on gas chromatography time-of-flight mass spectrometry (GC-TOFMS) at the University of Hawaii Cancer Center Metabolomics Shared Resource to establish an in-house metabolite database. Solvents, including methanol, acetonitrile, hexane, pyridine, and chloroform, were LC-MS grade or equivalent (Thermo Fisher Scientific). Ultrapure water is produced by a Milli-Q Reference system equipped with an LC-MS Pak filter (MilliporeSigma, Burlington, MA, USA) in the laboratory. The sample preparation procedures for GC-TOFMS were based on our previously published method with modifications [8]. Briefly, samples were spiked with internal standards and extracted with an appropriate organic solvent combination. Sample extracts were lyophilized and derivatized with methoxyamine and MSTFA to generate volatile TMS derivatives. Pooled samples containing aliquots from all the study subjects were used as study QCs and for correction of inter-batch analysis. Internal standards, standard mixture quality controls, and solvent blanks were analyzed and used to monitor data quality throughout the study. A GC-TOFMS system (LECO Corp., St. Joseph, MI, USA) was used for untargeted metabolomics profiling in mouse liver tissue samples. The optimized instrument settings are found in Table S2. Metabolite annotation was performed by comparing with reference standards in our in-house library (containing ~1200 endogenous metabolites). Commercial libraries, such as the National Institute of Standards and Technology (NIST) library 2010 and LECO/Fiehn Metabolomics Library for GC-TOFMS metabolome data (similarity threshold of 700 of 1000), were also used for validation and additional compound annotation.

### 2.6. Bioinformatics Analysis

Quality of Illumina sequence FASTQ files were explored using FASTQC prior to cleaning. The cleaning procedures of reads include trimming adapters and bases with Phred quality score less than 20 from both 3′ and 5′ ends using Cutadapt. Cleaned reads were imported into Partek Flow software (Partek Inc., St. Louis, MO, USA) to align against mm10 mouse reference genome using STAR and quantification to annotation model using Partek E/M. Gene counts were used for the differential gene expression analysis using DESeq2 Bioconductor package [9] as implemented in Partek Flow software and genes with FDR-adjusted *p*-values less than 0.05 were considered differentially expressed. Furthermore, functional and pathway analyses, as well as gene ontology (GO), ChIP enrichment analysis (ChEA) and kinase enrichment analysis (KEA) on the differentially expressed genes were done using Ingenuity Pathway Analysis (IPA; Qiagen), DAVID [10], and Enrichr [11,12] tools. For metabolomics and transcriptomics integration, the open-source MetaboAnalyst 4.0 software, maintained by the Xia Laboratory at McGill University (Canada), was used [13].

### 2.7. Western Blot

Livers were homogenized in CelLytic MT reagent (Sigma-Aldrich/MilliporeSigma) with protease inhibitors (Calbiochem/MilliporeSigma, Burlington, MA, USA) and subject to Western Blot analysis. Ten μg of total protein was loaded into 4–20% TGX gel in an SDS-PAGE chamber (BioRad, Hercules, CA, USA), electrophoresed, then transferred overnight to a PVDF membrane in Tris-glycine buffer containing 9% methanol. Quantification of protein expression was carried out in an Odyssey CLx Imaging System (Li-Cor Biosciences, Lincoln, NE, USA).

### 2.8. Amino Acid Assays

Glycine Assay Kit (Biovision, Milpitas, CA, USA) were used according to the manufacturer’s protocol to assess for glycine levels in 10 μL of serum and ~10 mg of liver tissue. Creatine levels were assessed using the Creatine Assay Kit (Sigma-Aldrich/MilliporeSigma), and following the protocol provided by the manufacturer.

### 2.9. Statistical Analysis

Power analysis for RNA-Seq was performed with RnaSeqSampleSize package [14] and for the metabolomic analysis using the MetaboAnalyst tool (http://www.metaboanalyst.ca). Statistical tests for non-omics approaches were performed rendering 0.05 as alpha using GraphPad Prism 7 (GraphPad Software Inc., San Diego, CA, USA), and mostly consisted of either Student’s *t*-test or two-way analysis of variance (two-way ANOVA) followed by Bonferroni’s ad hoc post-test.

## 3. Results

### 3.1. Effect of Scly Disruption on the Hepatic Transcriptome

**Figure 1:**
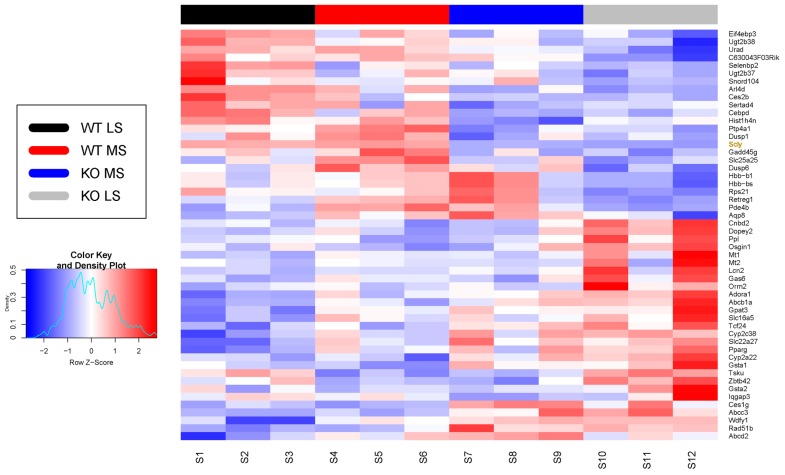
Heatmap of differentially expressed genes unveiled by RNA-Seq analysis with livers from Scly KO and WT mice fed diets containing 0.08 (mildly low) or 0.25 (adequate, medium) μg/g of sodium selenite. Density plot on the left represents z-scores. WT LS, wild-type low selenium; WT MS, wild-type medium selenium; KO LS, Scly KO low selenium; and KO MS, Scly KO medium selenium. *n* = 3 per experimental group.

RNA-Seq analysis revealed 52 genes to be differentially expressed in the Scly KO versus WT mice (Figure 1 and Supplementary Table S3), with most of the changes being accentuated by selenium deficiency in the Scly KO mice. Full RNA-Seq results can be found on the National Center for Biotechnology Information (NCBI) Gene Expression Omnibus (GEO) repository under Accession Number GSE137205.

Based on the gene function and relevance to the metabolic phenotype observed in this knockout mouse model [6], selected differentially expressed genes were validated by qPCR analysis according to most significant *p*-values in the RNA-Seq, regardless of whether up-or downregulated. Changes in expression were confirmed for eleven genes in male Scly KO mice according to either genotype or dietary selenium level (Table 1). Genes upregulated by Scly KO included ATP-binding cassette subfamily C member (*Abcc3*), ADP ribosylation factor-like 4D (*Arl4d*), and peroxisome proliferator-activated receptor gamma (*Pparg*), while downregulated genes included eukaryotic translation initiation factor 4E binding protein 3 (*Eif4ebp3*), inhibin beta-E (*Inhbe*), and selenium binding protein 2 (*Selenbp2*). Selenium levels regulated the expression of four genes independent of genotype, ATP-binding cassette subfamily B member 1A (*Abcb1a*), *Abcc3*, *Inhbe*, and periplakin (*Ppl*).

We previously reported significant sex differences in the metabolic phenotype of the Scly KO mouse model [15], and thus, expanded our validation analysis to include female Scly KO mice. We performed qPCR in female livers for the genes found to be affected in male Scly KO mice (Table 2). In females, Scly KO led to upregulation of the genes for *Abcb1a* and *Inhbe*, and downregulation of the genes for acyl-CoA thioesterase 3 (*Acot3*), lipocalin 2 (*Lcn2*), metallothionein 1 (*Mt1*), metallothionein 2 (*Mt2*), *Selenbp2*, and solute carrier family 25 member 25 (*Slc25a25*). Selenium levels affected only two genes, *Acot3* and *Arl4d*, in female mice.

Based on the fold-change differences observed for each gene from the RNA-Seq screening (raw data available at GEO Accession Number GSE137205), pathway analysis was performed. Using IPA, we found 42 pathways enriched in Scly KO versus WT mice; pathways that exhibited the highest enrichment are listed in Table 3. Using KEGG pathway analysis, we uncovered mineral absorption, ABC transporters and selenocompound metabolism as pathways with the highest combined score for differential activation in livers of Scly KO mice (Supplementary Table S4).

Using the Enrichr online tool [11,12], we performed gene ontology, kinase enrichment (KEA), ChIP enrichment (ChEA) analyses. Gene ontology analysis revealed ligand binding domain (LBD) as the molecular function with the highest score between WT and Scly KO mice fed a low selenium diet. Gene ontology of biological process revealed regulation of interleukin secretion to have the highest score between these same groups. KEA analysis uncovered cyclin-dependent kinase 9 (Cdk9) and mitogen-activated protein kinase kinase 1 (MAP2K1) as upstream kinases possibly regulating the formation of the subnetwork. Interestingly, ChEA analysis revealed CCAT enhancer binding protein alpha (CEBPα), peroxisome proliferator-activated receptor alpha (PPARα), retinoid X receptor (RXR), liver X receptor (LXR) and PPARγ as possible upstream transcription factors leading to the observed metabolic differences between WT and Scly KO mice (Supplementary Table S4).

### 3.2. Sex Differences in Hepatic Metabolites of Scly KO Mice

**Figure 2:**
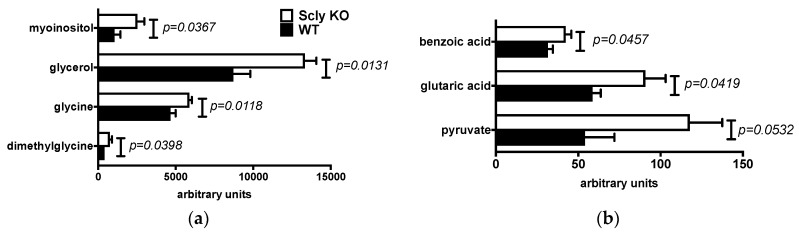

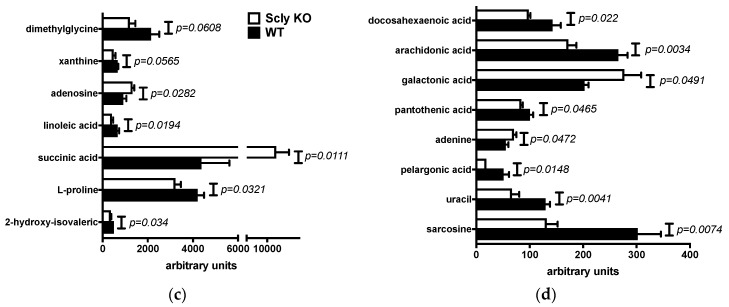
Differentially available metabolites in the livers of male (**a**,**b**) and female (**c**,**d**) mice fed a selenium-adequate diet. (**a**,**c**) are metabolites found in higher levels; (**b**,**d**) are metabolites found in lower levels. WT, wild-type, black bars; KO, knockout, white bars. Values are mean + SEM, *n* = 5 per group. *p* values were calculated after the Student’s *t*-test with alpha = 0.05.

The physiological effects of disruption of selenium recycling have been investigated [6], but not in the context of overall changes in the metabolome. We pursued metabolomics analysis of the livers of male and female WT, and Scly KO mice fed a selenium-adequate diet, and detailed metabolomics results are found in the Supplementary Table S5. In males, seven metabolites were elevated by the disruption of the *Scly* gene (Figure 2a,b). In addition, pyruvate presented a non-significant statistical trend towards higher levels, with a *p*-value of 0.0532 (Figure 2b).

Strikingly, in female Scly KO mice, however, thirteen metabolites were found to vary in their livers significantly (Figure 2c,d). Moreover, two additional metabolites, dimethylglycine and xanthine, presented *p*-values of 0.0608 and 0.0565 respectively, pointing to a trend towards decreased levels of these metabolites in the Scly KO mice (Figure 2c). This result was in contrast with males. Compared to the thirteen differentially detected metabolites in female Scly KO mouse livers, with the majority in lower levels in the Scly KO mice, the majority of metabolites were increased in males.

### 3.3. Pathway Enrichment Analysis of the Livers of Scly KO Mice

*In silico*, we combined results of the transcriptomics and metabolomics analysis of male mice fed adequate selenium and performed an integrated omics analysis, using the MetaboAnalyst 4.0 software package and the metabolites listed in Supplementary Table S6. Integrated omics revealed pathways to be affected by the absence of the *Scly* in the livers of mice. These pathways are disclosed in the Supplementary Table S7, with the top 10 enriched pathways compiled in Table 4. The most enriched pathway was alanine, aspartate and glutamate metabolism. Interestingly, five amino acid pathways were enriched in the analysis, plus the aminoacyl-tRNA biosynthesis pathway, which charges tRNAs with amino acids for protein translation. 

### 3.4. Glycine Metabolism in the Scly KO Mice

Dimethylglycine levels changed both in males and females as described in Section 3.2, but in different directions. Male Scly KO mice had increased levels of dimethylglycine in their livers, while female Scly KO mice had a trend towards lower levels of this metabolite. Dimethylglycine is the precursor for the synthesis of sarcosine and glycine, two metabolites that also changed in livers of Scly KO mice.

**Figure 3:**
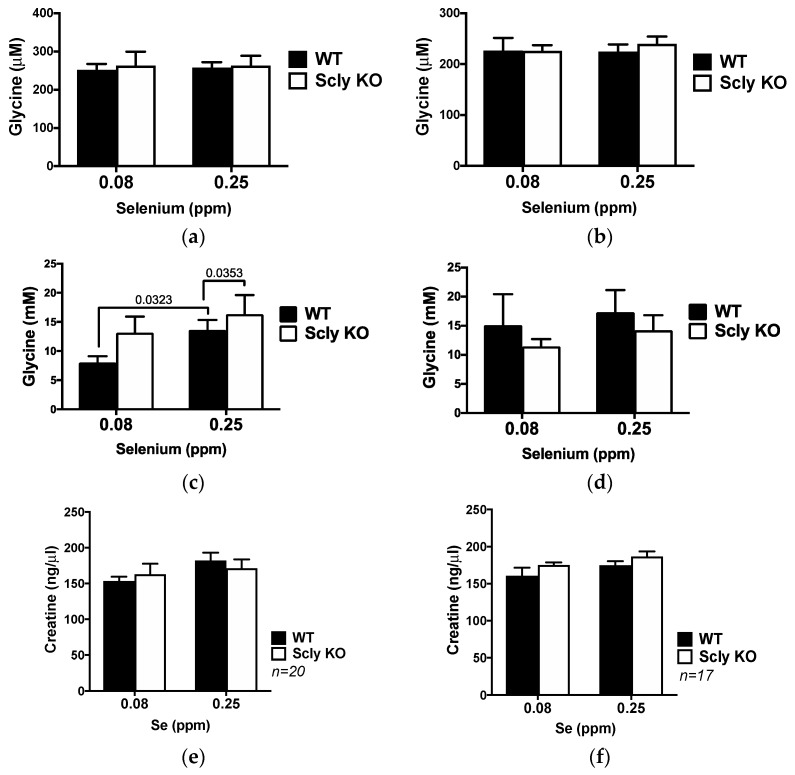
Glycine and creatine levels in male (**a**,**c**,**e**) and female (**b**,**d**,**f**) mice fed selenium-deficient and selenium-adequate diets; (**a**,**b**) represent levels of glycine in the serum, (**c**,**d**) show levels of glycine in the liver, (**e**,**f**) represent levels of creatine in the liver. Black bars, WT; white bars, Scly KO mice; *p* values are displayed in graphs when they reach ≤0.05. Data are means + SEM, *n* = 8 for WT and *n* = 7 for Scly KO mice for glycine measurements.

Paradoxically, while males had a slight, but statistically significant, elevation of glycine, females had decreased levels of sarcosine. Dimethylglycine conversion into sarcosine is catalyzed by the enzyme dimethylglycine dehydrogenase (DMGDH), and sarcosine availability is dependent on the levels of the amino acid creatine [16]. We assessed levels of glycine in the liver and in the serum (Figure 3a–d), and of creatine in the liver (Figure 3e,f) of WT and Scly KO mice. Serum glycine levels in both sexes were unchanged (Figure 3a,b). Nevertheless, only male Scly KO mice had increased levels of glycine in their livers (Figure 3c). Female Scly KO mice maintained hepatic glycine (Figure 3d) at the same level as their WT counterparts. Hepatic creatine levels in both sexes, genotypes and diets were unchanged (Figure 3e,f).

**Figure 4:**
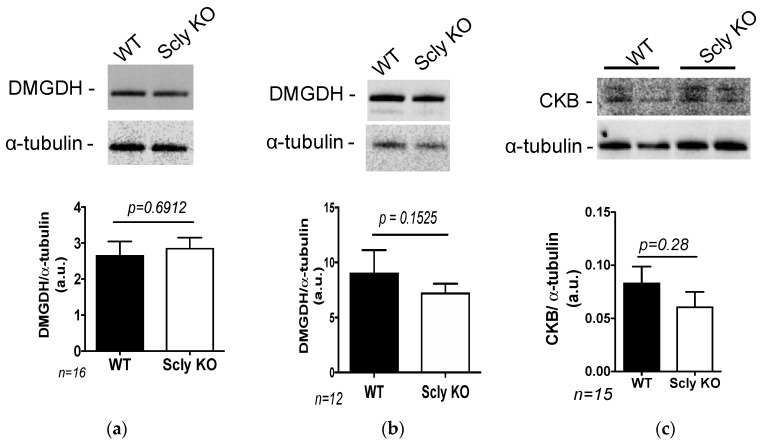
Hepatic expression of enzymes involved in glycine metabolism in male mice. (**a**) DMGDH expression in mice fed a selenium-deficient. (**b**) DMGDH expression in mice fed a selenium-adequate diet. (**c**) CKB expression in mice fed a selenium-adequate diet. All results were normalized by expression levels of α-tubulin. Data are means ± SEM, after the Student’s *t*-test with alpha = 0.05; sample number is displayed in the graphs.

We additionally tested whether the expression of DMGDH was affected in these animals. To our surprise, DMGDH expression was maintained in male Scly KO mice livers, regardless of selenium levels in the diet (Figure 4a,b). Moreover, the expression of creatine kinase B (CKB), the hepatic enzyme responsible for creatine synthesis, was unchanged in male Scly KO mice fed a selenium-adequate diet compared to WT mice (Figure 4c).

## 4. Discussion

The physiological role of SCL in vertebrates has been mostly analyzed and discussed in the context of its action on selenium metabolism, specifically its well-characterized biochemical function of selenocysteine decomposition as a source of selenide for selenoprotein synthesis [4,17,18]. Nevertheless, the potential relationship of SCL-dependent selenocysteine decomposition with additional molecular pathways has been sparsely investigated, the sole report of a regulatory function being that of providing 2-naphtol to major urinary proteins [19]. Using the Scly KO mouse model and a combined approach with transcriptomics and metabolomics analyses, we identified hepatic molecular pathways, particularly involving the metabolism of several amino acids, that are affected by the disruption of selenocysteine decomposition in mice. The Scly KO mice presents a metabolic syndrome-like phenotype when fed a selenium-adequate diet. In these conditions, selenoprotein gene expression is elevated, but selenoprotein levels are mostly maintained in the liver, where mSCL is most active. Moreover, we previously observed enrichment of pyruvate in the livers of male Scly KO mice [7], a finding further confirmed in our metabolomic results presented here. The metabolic consequences of the absence of *Scly* suggest the involvement of mSCL in controlling aspects of hepatic energy metabolism that may go beyond selenoprotein levels and its actions.

Our RNA-Seq analysis revealed the extent to which *Scly* absence predominantly affects transcription factors. Intriguingly, the most enriched pathway uncovered is pregnane and xenobiotic receptor/ retinoid X receptor (PXR/RXR) activation. These two transcription factors regulate the expression of genes involved in xenobiotic metabolism in nonalcoholic fatty liver disease [20]. Combined with the observed upregulation of *Pparg*, a classic coordinator of lipogenesis and lipid metabolism [21], our PXR/RXR results in the Scly KO mouse liver suggest that these two nuclear regulatory pathways may be involved in the lipid deposition observed in Scly KO mice hepatocytes. A ChEA analysis with the RNA-Seq results confirmed this possibility, as several transcription factors known to be involved in the control of hepatic lipid metabolism, such as CEBPα, PPARα, PPARγ, RXR, and LXR [20,22,23], obtained high combined scores in this analysis. Moreover, gene ontology also confirmed RXR as a potential link that can explain the metabolic phenotype of the Scly KO mice. Further studies to establish the molecular mechanism for the activation of this transcription factor specifically in the Scly KO mouse liver may enrich our understanding of the connection between selenium and energy metabolism that leads to the metabolic syndrome phenotype of this mouse model.

Still, these effects on nuclear factors can be consequences of a primary effect that SCL has on hepatic amino acid metabolism. Localized selenium deficiency could be more relevant to the observed changes in amino acid pathways in the Scly KO mice, as metabolomic profiling combined with transcriptomics performed in the liver of selenium-deficient mice also revealed increases in amino acid levels and significant enrichment of amino acid pathways [24]. Unsurprisingly, disruption of *Scly* slightly decreases selenium content in the liver [6], which could explain the observed effect on amino acid pathways.

Selenium-deficient mice also had significant changes in amino acid pathways in the serum [25], galvanizing these pathways as responsive to low selenium levels. Specifically, the study mentioned also showed that serum glycine levels were enhanced in selenium deficiency, an effect that we did not observe in the serum of Scly KO mice. Serum glycine has been negatively associated with body mass index, insulin resistance and triacylglycerol in humans [26]. The Scly KO mice, however, were obese and insulin resistant under selenium-adequate conditions. This obesity state worsened by selenium deficiency [6], without changes in serum glycine or hepatic creatine, a product of glycine utilization [16]. Nevertheless, glycine levels were elevated in the liver of the Scly KO mice. This paradoxical result points to a regulatory, indirect involvement of SCL in determining glycine levels in the liver. Such an effect could occur either via consumption of hepatic glycine to synthesize glutathione, ameliorating oxidative stress, or via a modulatory role enhancing the uptake of glycine for hepatic gluconeogenesis, as these coordinate the fate of glycine in the liver [27]. In both cases, less glycine would be released to the bloodstream. Also, the effect of selenium status on glycine levels could be mediated by a selenoprotein synthesized with the participation of SCL. Neither of these possibilities has been explored yet. Selenium deficiency, which upregulates *Scly* [5] and downregulates selenoproteins curbing ROS [28], increases circulating glycine [25], while selenoprotein deficiency in mice leads to a type 2 diabetes-like phenotype [29]. Without *Scly*, selenoprotein production is further dampened, and our results uncovered that amino acid pathways are the ones most affected, with these effects possibly leading to the observed metabolic issues.

Interestingly, glycine can also be converted to serine, a reaction catalyzed by serine hydroxymethyltransferase 1, an enzyme positively regulated by selenium [30]. Selenium-deficient mice were reported to have elevated circulating glycine, serine, and pyruvate [25]. Moreover, a single nucleotide polymorphism in the gene for DMGDH, an enzyme involved in glycine synthesis using the precursor dimethylglycine, has been associated with selenium status in humans [31]. To our surprise, however, the hepatic expression of DMGDH was maintained in the Scly KO mice, pointing possibly to a downstream modulator of glycine affected by SCL. In contrast, the enzyme glycine-*N*-methyltransferase (GNMT), responsible for glycine synthesis and downstream from DMGDH, is upregulated by selenite supplementation in the liver of male mice [32]. Hence, the paradox between selenium, SCL and glycine metabolism warrants further investigation.

Lastly, we observed striking differences in hepatic transcriptomic and metabolite profiling between male and female Scly KO mice. Selenium metabolism as a whole is sexually dimorphic [33,34], and these sex differences in the phenotype of the Scly KO mouse model have been previously discussed [15]. Hence, it is not surprising to uncover that in our analysis. The specific molecular mechanisms responsible for the sex differences in the liver of the Scly KO mouse are still not fully understood, and these results help unwind pieces of this puzzle. Still, future studies on the intricacies of the regulation of SCL by sex-dependent factors could improve our understanding of selenium metabolism and energy metabolism as well.

## 5. Conclusions

Using a combined transcriptomics and metabolomics approach, we identified alterations in hepatic amino acid metabolism after the disruption of the *Scly* gene in male mice. Additionally, we uncovered glycine as a differential amino acid elevated in the livers of Scly KO mice in a sex-dependent manner. These results may provide future insights into the molecular mechanisms by which SCL regulates amino acid levels in the liver, potentially connecting selenium utilization with energy metabolism.

## Figures and Tables

**Table 1:** Validation by qPCR analysis of differentially expressed transcripts in livers of male Scly KO versus WT mice fed selenium-deficient (0.08 μg/g) and selenium-adequate (0.25 μg/g) diets.

Gene	0.08 g/g Se		0.25 g/g Se		2-Way ANOVA		
	WT (*n* = 5)	Scly KO (*n* = 5)	WT (*n* = 4)	Scly KO (*n* = 4)	*p*_interaction_	*p*_Se_	*p*_genotype_
*Abcb1a*	0.403 ± 0.016	0.302 ± 0.052	0.43 ± 0.038	0.571 ± 0.06	***0.0193***	***0.0063***	0.6606
*Abcc3*	0.556 ± 0.034	0.498 ± 0.077	0.476 ± 0.235	1.205 ± 0.143	***0.0003***	***0.0019***	***0.0012***
*Acot3*	0.402 ± 0.177	0.278 ± 0.205	0.526 ± 0.384	0.638 ± 0.190	0.3654	0.0783	0.9596
*Angptl6*	0.56 ± 0.049	0.501 ± 0.127	0.622 ± 0.172	0.584 ± 0.2	0.8866	0.3468	0.5221
*Arl4d*	0.86 ± 0.287	0.858 ± 0.092	0.408 ± 0.135	1.275 ± 0.604	***0.0321***	0.9247	***0.0329***
*Cesg1*	5.394 ± 0.903	3.382 ± 0.158	2.399 ± 0.873	4.382 ± 1.778	***0.0043***	0.1055	0.9801
*Eif4ebp3*	1.118 ± 0.234	0.436 ± 0.108	1.482 ± 0.4	0.518 ± 0.198	0.316	0.123	***<0.0001***
*Inhbe*	3.332 ± 0.484	1.187 ± 0.241	3.815 ± 0.739	2.297 ± 0.454	0.2633	***0.0114***	***<0.0001***
*Lcn2*	0.428 ± 0.297	0.348 ± 0.099	0.642 ± 0.357	0.426 ± 0.187	0.6491	0.3353	0.3292
*Mt1*	0.709 ± 0.322	0.133 ± 0.044	0.201 ± 0.096	0.304 ± 0.207	***0.01***	0.1562	0.0551
*Mt2*	0.688 ± 0.292	0.177 ± 0.054	0.25 ± 0.112	0.344 ± 0.13	***0.0088***	0.1869	0.0524
*Pparg*	0.166 ± 0.052	0.692 ± 0.103	0.193 ± 0.082	0.683 ± 0.087	0.8335	0.9209	***<0.0001***
*Ppl*	0.089 ± 0.022	0.112 ± 0.059	0.151 ± 0.03	0.196 ± 0.028	0.5362	***0.0011***	0.0754
*Selenbp2*	13.92 ± 7.954	6.691 ± 1.122	20.88 ± 10.12	6.224 ± 1.178	0.3281	0.3504	***0.008***
*Slc25a25*	6.697 ± 0.888	3.163 ± 0.958	6.147 ± 3.191	4.869 ± 0.781	0.1732	0.5319	0.0357

WT, wild-type; KO, knockout. Sample size of each experimental group is indicated in the table. Values are means ± SEM. Bold and italic represent statistically significant values after two-way analysis of variance (ANOVA), rendering alpha = 0.05.

**Table 2:** Validation by qPCR analysis of differentially expressed transcripts in livers of female Scly KO and WT mice fed selenium-deficient (0.08 μg/g) and selenium-adequate (0.25 μg/g).

Gene	0.08 g/g Se		0.25 g/g Se		2-Way ANOVA		
	WT (*n* = 4)	Scly KO (*n* = 5)	WT (*n* = 4)	Scly KO (*n* = 5)	*p*_interaction_	*p*_Se_	*p*_genotype_
*Abcb1a*	0.579 ± 0.203	1.065 ± 0.503	0.727 ± 0.265	1.77 ± 0.707	0.2541	0.0914	***0.0065***
*Abcc3*	1.075 ± 0.578	1.496 ± 0.741	1.401 ± 0.605	1.673 ± 0.556	0.8063	0.4129	0.2647
*Acot3*	0.251 ± 0.043	0.114 ± 0.066	0.124 ± 0.029	0.106 ± 0.057	***0.0323***	***0.0163***	***0.0074***
*Angptl6*	0.012 ± 0.002	0.014 ± 0.007	0.009 ± 0.001	0.016 ± 0.006	0.3646	0.7616	0.0713
*Arl4d*	0.561 ± 0.235	0.382 ± 0.171	1.147 ± 0.662	0.852 ± 0.541	0.7996	***0.0345***	0.3081
*Cesg1*	4.006 ± 1.288	4.223 ± 1.249	4.339 ± 0.751	5.236 ± 1.125	0.537	0.231	0.3175
*Eif4ebp3*	0.016 ± 0.008	0.017 ± 0.0146	0.011 ± 0.003	0.016 ± 0.007	0.6982	0.5723	0.5054
*Inhbe*	1.899 ± 0.719	3.481 ± 0.845	2.338 ± 0.949	4.11 ± 0.961	0.8286	0.2351	***0.0018***
*Lcn2*	0.026 ± 0.01	0.017 ± 0.006	0.025 ± 0.009	0.016 ± 0.005	0.9079	0.7187	***0.024***
*Mt1*	0.314 ± 0.092	0.154 ± 0.12	0.205 ± 0.124	0.073 ± 0.062	0.7741	0.0696	***0.0092***
*Mt2*	0.397 ± 0.135	0.186 ± 0.121	0.259 ± 0.18	0.1 ± 0.063	0.6717	0.0844	***0.0082***
*Pparg*	0.524 ± 0.246	0.465 ± 0.234	0.753 ± 0.165	0.6 ± 0.208	0.6549	0.098	0.319
*Ppl*	0.004 ± 0.003	0.003 ± 0.002	0.003 ± 0.002	0.003 ± 0.001	0.5335	0.6621	0.4059
*Selenbp2*	0.122 ± 0.048	0.069 ± 0.038	0.077 ± 0.022	0.057 ± 0.017	0.3098	0.0889	***0.0338***
*Slc25a25*	0.209 ± 0.064	0.043 ± 0.013	0.087 ± 0.054	0.111 ± 0.06	***0.0015***	0.27	***0.0109***

WT, wild-type; KO, knockout. Sample size of each experimental group is indicated in the table. Values are mean ± SEM, Bold and italic represent statistically significant values after Two-way ANOVA, rendering alpha = 0.05.

**Table 3:** Biological pathways exhibiting highest transcript enrichment in Scly KO versus WT mice livers according to IPA.

Ingenuity Canonical Pathways	−log (*p*-Value)	Ratio
PXR/RXR Activation	4.11	0.0462
LPS/IL-1 Mediated Inhibition of RXR Function	3.76	0.018
Xenobiotic Metabolism Signaling	3.32	0.0138
Nicotine Degradation III	2.65	0.0364
Melatonin Degradation I	2.52	0.0312
Nicotine Degradation II	2.52	0.0312
Superpathway of Melatonin Degradation	2.45	0.029
Acyl-CoA Hydrolysis	1.82	0.0833
Hepatic Cholestasis	1.75	0.0125
Ubiquinol-10 Biosynthesis (Eukaryotic)	1.62	0.0526

**Table 4:** Top 10 biological pathways enriched by the disruption of Scly according to integrated omics analysis.

Biological Pathways	Hits	*p*-Value
Alanine, aspartate and glutamate metabolism	10	0.000285
D-glutamine and D-glutamate metabolism	4	0.000299
Galactose metabolism	9	0.000886
Aminoacyl-tRNA biosynthesis	12	0.000907
Biosynthesis of unsaturated fatty acids	6	0.002
Pantothenate and CoA biosynthesis	5	0.021
Valine, leucine and isoleucine biosynthesis	3	0.022
Glyoxylate and dicarboxylate metabolism	6	0.045
Taurine and hypotaurine metabolism	3	0.051
Glycine, serine and threonine metabolism	7	0.053

## Supplementary Materials

The following are available online at https://www.mdpi.com/2072-6643/11/11/2584/s1, Table S1: Mouse primers used in the qPCR analysis. Table S2: GC-TOF MS instrument settings. Table S3: Differentially expressed genes in livers of male WT and Scly KO mice fed an adequate selenium diet. Table S4: Gene ontology, ChIP enrichment analysis (ChEA), kinase enrichment analysis (KEA), and KEGG Pathway analysis of male WT and Scly KO mice. Table S5: Metabolites enriched in the livers of Scly KO mice versus WT mice on an adequate selenium diet. Table S6: Metabolite list from Table S5 with fold changes, used in the MetaboAnalyst analysis. Table S7: MetaboAnalyst Joint Pathway results. Raw RNA-Seq data from this paper is available at the NCBI GEO repository and can be accessed with the Accession Number GSE137205.

## References

[1] Labunskyy V.M., Hatfield D.L., Gladyshev V.N. (2014). Selenoproteins: Molecular pathways and physiological roles. Physiol. Rev..

[2] Ha H.Y., Alfulaij N., Berry M.J., Seale L.A. (2019). From Selenium Absorption to Selenoprotein Degradation. Biol. Trace Elem. Res..

[3] Esaki N., Karai N., Nakamura T., Tanaka H., Soda K. (1985). Mechanism of reactions catalyzed by selenocysteine beta-lyase. Arch. Biochem. Biophys..

[4] Kurokawa S., Takehashi M., Tanaka H., Mihara H., Kurihara T., Tanaka S., Hill K., Burk R., Esaki N. (2011). Mammalian selenocysteine lyase is involved in selenoprotein biosynthesis. J. Nutr. Sci. Vitam..

[5] Seale L.A., Ha H.Y., Hashimoto A.C., Berry M.J. (2018). Relationship between selenoprotein P and selenocysteine lyase: Insights into selenium metabolism. Free Radic. Biol. Med..

[6] Seale L.A., Gilman C.L., Hashimoto A.C., Ogawa-Wong A.N., Berry M.J. (2015). Diet.-Induced Obesity in the Selenocysteine Lyase Knockout Mouse. Antioxid. Redox Signal..

[7] Seale L.A., Hashimoto A.C., Kurokawa S., Gilman C.L., Seyedali A., Bellinger F.P., Raman A.V., Berry M.J. (2012). Disruption of the selenocysteine lyase-mediated selenium recycling pathway leads to metabolic syndrome in mice. Mol. Cell. Biol..

[8] Pan L., Qiu Y.P., Chen T.L., Lin J.C., Chi Y., Su M.M., Zhao A.H., Jia W. (2010). An optimized procedure for metabonomic analysis of rat liver tissue using gas chromatography/time-of-flight mass spectrometry. J. Pharm. Biomed. Anal..

[9] Love M.I., Huber W., Anders S. (2014). Moderated estimation of fold change and dispersion for RNA-seq data with DESeq2. Genome Biol..

[10] Huang D.W., Sherman B.T., Tan Q., Kir J., Liu D., Bryant D., Guo Y.J., Stephens R., Baseler M.W., Lane H.C. (2007). DAVID Bioinformatics Resources: Expanded annotation database and novel algorithms to better extract biology from large gene lists. Nucleic Acids Res..

[11] Chen E.Y., Tan C.M., Kou Y., Duan Q.N., Wang Z.C., Meirelles G.V., Clark N.R., Ma’ayan A. (2013). Enrichr: Interactive and collaborative HTML5 gene list enrichment analysis tool. BMC Bioinform..

[12] Kuleshov M.V., Jones M.R., Rouillard A.D., Fernandez N.F., Duan Q.N., Wang Z.C., Koplev S., Jenkins S.L., Jagodnik K.M., Lachmann A. (2016). Enrichr: A comprehensive gene set enrichment analysis web server 2016 update. Nucleic Acids Res..

[13] Chong J., Soufan O., Li C., Caraus I., Li S., Bourque G., Wishart D.S., Xia J. (2018). MetaboAnalyst 4.0: Towards more transparent and integrative metabolomics analysis. Nucleic Acids Res..

[14] Zhao S., Li C.I., Guo Y., Sheng Q., Shyr Y. (2018). RnaSeqSampleSize: Real data based sample size estimation for RNA sequencing. BMC Bioinform..

[15] Ogawa-Wong A.N., Hashimoto A.C., Ha H., Pitts M.W., Seale L.A., Berry M.J. (2018). Sexual Dimorphism in the Selenocysteine Lyase Knockout Mouse. Nutrients.

[16] Wang W., Wu Z., Dai Z., Yang Y., Wang J., Wu G. (2013). Glycine metabolism in animals and humans: Implications for nutrition and health. Amino Acids.

[17] Tobe R., Mihara H. (2018). Delivery of selenium to selenophosphate synthetase for selenoprotein biosynthesis. Biochim. Biophys. Acta Gen. Subj..

[18] Mihara H., Esaki N., Hatfield D.L., Gladyshev V.N., Berry M.J. (2012). Selenocysteine lyase: Mechanism, structure, and biological role. Selenium—Its Molecular Biology and Role in Human Health.

[19] Kwak M.S., Mihara H., Esaki N. (2003). A novel regulatory function of selenocysteine lyase, a unique catalyst to modulate major urinary protein. J. Mol. Catal. B Enzym..

[20] Cave M.C., Clair H.B., Hardesty J.E., Falkner K.C., Feng W., Clark B.J., Sidey J., Shi H., Aqel B.A., McClain C.J. (2016). Nuclear receptors and nonalcoholic fatty liver disease. Biochim. Biophys. Acta Gene Regul. Mech..

[21] Semple R.K., Chatterjee V.K., O‘Rahilly S. (2006). PPAR gamma and human metabolic disease. J. Clin. Investig..

[22] Liss K.H., Finck B.N. (2017). PPARs and nonalcoholic fatty liver disease. Biochimie.

[23] Wang Y., Viscarra J., Kim S.J., Sul H.S. (2015). Transcriptional regulation of hepatic lipogenesis. Nat. Rev. Mol. Cell Biol..

[24] Yim S.H., Clish C.B., Gladyshev V.N. (2019). Selenium Deficiency Is Associated with Pro-Longevity Mechanisms. Cell Rep..

[25] Mickiewicz B., Villemaire M.L., Sandercock L.E., Jirik F.R., Vogel H.J. (2014). Metabolic changes associated with selenium deficiency in mice. Biometals Int. J. Role Met. Ions Biol. Biochem. Med..

[26] Gar C., Rottenkolber M., Prehn C., Adamski J., Seissler J., Lechner A. (2018). Serum and plasma amino acids as markers of prediabetes, insulin resistance, and incident diabetes. Crit. Rev. Clin. Lab. Sci..

[27] Alves A., Bassot A., Bulteau A.L., Pirola L., Morio B. (2019). Glycine Metabolism and Its Alterations in Obesity and Metabolic Diseases. Nutrients.

[28] Sunde R.A., Raines A.M., Barnes K.M., Evenson J.K. (2009). Selenium status highly regulates selenoprotein mRNA levels for only a subset of the selenoproteins in the selenoproteome. Biosci. Rep..

[29] Labunskyy V.M., Lee B.C., Handy D.E., Loscalzo J., Hatfield D.L., Gladyshev V.N. (2011). Both maximal expression of selenoproteins and selenoprotein deficiency can promote development of type 2 diabetes-like phenotype in mice. Antioxid. Redox Signal..

[30] Speckmann B., Schulz S., Hiller F., Hesse D., Schumacher F., Kleuser B., Geisel J., Obeid R., Grune T., Kipp A.P. (2017). Selenium increases hepatic DNA methylation and modulates one-carbon metabolism in the liver of mice. J. Nutr. Biochem..

[31] Mao J., Vanderlelie J.J., Perkins A.V., Redman C.W., Ahmadi K.R., Rayman M.P. (2016). Genetic polymorphisms that affect selenium status and response to selenium supplementation in United Kingdom pregnant women. Am. J. Clin. Nutr..

[32] Lennicke C., Rahn J., Kipp A.P., Dojcinovic B.P., Muller A.S., Wessjohann L.A., Lichtenfels R., Seliger B. (2017). Individual effects of different selenocompounds on the hepatic proteome and energy metabolism of mice. Biochim. Biophys. Acta.

[33] Schomburg L., Hatfield D.L., Schweizer U., Tsuji P.A., Gladyshev V.N. (2016). Sex-specific differences in biological effects and metabolism of selenium. Selenium—Its Molecular Biology and Role in Human Health.

[34] Seale L.A., Ogawa-Wong A.N., Berry M.J. (2018). Sexual Dimorphism in Selenium Metabolism and Selenoproteins. Free Radic. Biol. Med..

